# Construction of a high-density genetic map and QTL mapping for pearl quality-related traits in *Hyriopsis cumingii*

**DOI:** 10.1038/srep32608

**Published:** 2016-09-02

**Authors:** Zhi-Yi Bai, Xue-Kai Han, Xiao-Jun Liu, Qing-Qing Li, Jia-Le Li

**Affiliations:** 1 Key Laboratory of Freshwater Aquatic Genetic Resources, Shanghai Ocean University, Ministry of Agriculture, Shanghai 201306, China; 2 Shanghai Engineering Research Centre of Aquaculture, Shanghai Ocean University, Shanghai 201306, China; 3 Aquaculture Division, E-Institute of Shanghai Universities, Shanghai Ocean University, Shanghai 201306, China

## Abstract

A high-density genetic map is essential for quantitative trait locus (QTL) fine mapping. In this study, 4,508 effective single nucleotide polymorphism markers (detected using specific-locus amplified fragment sequencing) and 475 microsatellites were mapped to 19 linkage groups (LGs) using a family with 157 individuals. The map spanned 2,713 cM, with an average of 259 markers and 79 loci per LG and an average inter-marker distance of 1.81 cM. To identify QTLs for pearl quality traits, 26 putatively significant QTLs were detected for 10 traits, including, three for shell width, seven for body weight, two for shell weight, two for margin mantle weight, five for inner mantle weight, and seven for shell nacre colour. Among them, five QTLs associated with shell nacre colour were mapped to LG17 and explained 19.7% to 22.8% of the trait variation; this suggests that some important genes or loci determine shell nacre colour in LG17. The linkage map and mapped QTLs for shell nacre colour would be useful for improving the quality of *Hyriopsis cumingii* via marker-assisted selection.

The triangle sail mussel, *Hyriopsis cumingii*, is widely distributed in large lakes, rivers, and estuaries in China. Nearly 95% of freshwater pearls worldwide are produced in China, of which about 70% are produced by *H. cumingii*^1^. Currently, modern *H. cumingii* aquaculture has several issues such as degradation of germplasm resources and decline in pearl quality. Therefore, it is important to genetically improve *H. cumingii*. Although traditional breeding has played a crucial role in genetic improvement, it relies mainly on phenotype, which is easily affected by environmental factors^2^. Nowadays, marker-assisted selection (MAS) provides a very efficient tool for selective breeding based on genotype. Unlike traditional selective breeding based on phenotype, MAS offers precise and rapid selection of the targeted gene and accelerates breeding. Development of effective genes or loci that affect traits is a precondition for MAS. Karyotype analysis has shown that *H. cumingii* has 38 chromosomes (19 pairs)^3^, and its genome size has been estimated to be approximately 3 Gb on the basis of k-mer analysis. The larger genome size, higher heterozygosity, and redundant sequences resulted in a lag in developing gene or marker resources for *H. cumingii*. Rapid development of sequencing techniques provided us with a great opportunity to produce valuable genomic resources for *H. cumingii*.

Quantitative trait locus (QTL) mapping is an effective method for identifying markers associated with economic traits for use in MAS, and construction of genetic linkage maps is a decisive precondition^4^,^5^. To date, linkage maps have been constructed for more than 40 aquaculture species, and QTL mapping has been performed for over 20 aquaculture species^6^. Nevertheless, QTL mapping has not been widely used for genetic breeding of aquaculture species. A key reason is that most first-generation genetic maps for aquaculture species have a limited number of amplified fragment length polymorphisms, restricted fragment length polymorphisms, or simple sequence repeats (SSRs); these maps have a low density, making it difficult to perform fine-scale mapping to narrow down genomic regions strongly associated with important traits. Therefore, a sufficient number of high-quality molecular markers are crucial for constructing a high-density linkage map and determining the efficiency and veracity of the genetic map^7^.

Single nucleotide polymorphism (SNP) is a variation in just one nucleotide at the genome level^8^. When compared with previous molecular markers, SNPs possess advantages such as high abundance in the genome and hereditable stability. In particular, SNPs are suitable for automated large-scale genotyping, allowing for genotyping of a large number of cost-effective markers to construct high-density genetic maps^9^. With the rapid development of sequencing techniques in the past decade, especially next-generation sequencing (NGS) techniques, various genotyping methods have been developed and thousands of SNPs were identified in many species. Currently, more than 30 different methods are being used for SNP genotyping^10^. For instance, restriction site-associated sequencing (RAD-seq) is being widely used because of its rapidity and reliability in genotyping and polymorphism identification^11^, and several methods with simpler library preparation protocols, such as 2b-RAD^12^ and double digest RAD-seq^13^, have been developed. Elshire *et al.*^14^ introduced an efficient genotyping-by-sequencing approach that can simultaneously detect and score thousands of SNPs. Besides genotyping based on NGS, SNPs are usually genotyped with SNP arrays. Some SNPs arrays have been developed and used for aquaculture species, such as the Atlantic salmon^15^,catfish^16^, common carp^17^, Atlantic cod^18^, and rainbow trout^19^. Because of these effective approaches, high-density SNP-based linkage maps have been constructed for several aquaculture species, such as the Atlantic salmon^20^, rainbow trout^21^, channel catfish^22^, Japanese flounder^23^, and Zhikong scallop^7^. Recently, a more effective method called specific-locus amplified fragment sequencing (SLAF-seq) was proposed by Sun *et al.*^24^. SLAF-seq is used a pre-designed reduced representation strategy and deep sequencing to improve the efficiency and accuracy of genotyping. Moreover, it provides a double barcode system that can be applied to large populations and is needed to construct the SLAF-seq library. After high-throughput sequencing, a large number of sequences were collected and used to obtain polymorphism SLAF tags by software alignment; then, specific SNP sites were found on the basis of these polymorphism SLAF tags. Thus, SLAF and SNP markers obtained via this approach are essentially the same. This technology has been applied for the construction of high-density linkage maps for several species, such as cucumber^25^, soybean^26^, common carp^24^, and tea^27^.

Since more genetic maps have been constructed for bivalves, QTL mapping for some important economic traits has been performed recently. For instance, QTL mapping for growth traits and disease resistance has been conducted in the Pacific oyster^28^,^29^; QTL mapping for growth and shell colour has been conducted in *Argopecten irradians*^30^,^31^; and QTL mapping for growth traits and sex-determination has been conducted in *Chlamys farreri*^7^. In addition, for some pearl mussels, QTLs have been identified for growth and shell colour in the freshwater mussel *H. cumingii*^32^, and QTLs for pearl quality and shell growth have been identified in *Pinctada maxima*^33^,^34^. The identified QTLs will provide the basis for MAS of important economic traits in these bivalves.

In this study, we used 157 F_1_ individuals to perform whole-genome genotyping with SLAF-seq and constructed a high-density genetic map for *H. cumingii*. Furthermore, we performed QTL screening for pearl quality-related traits by using this map. The high-density genetic map will provide an essential tool for QTL fine mapping and identifying candidate genes associated with complex traits. Identification of QTLs related to pearl quality traits may remarkably facilitate breeding programs in the future.

## Results

### Analysis of SLAF-seq data for 157 F_1_ individuals and two parents

In this study, we first constructed the SLAF-seq library for *H. cumingii* with 157 F_1_ individuals and two parents. Using high-throughput sequencing and bioinformatics, a total of 139,113,148 pair-end reads were obtained for the SLAF library. Among them, 88.64% bases were of a high quality (Q score > 30), and GC content was 37.45% on average. The number of reads for male and female parents was 9,025,115 and 8,571,660, respectively. For the F_1_ population, read numbers ranged from 523,546 to 1,009,487 (average, 773,990; Table S1).

All reads were clustered into SLAFs on the basis of sequence similarity. After correcting or discarding low-depth SLAF tags, 239,704 high-quality SLAFs were identified, of which 201,805 and 206,806 were detected in the male and female parents, respectively. The coverage of SLAFs for paternal and maternal parents was 19.25-fold and 20.70-fold, respectively. For the F_1_ population, 62,790 to 140,362 SLAFs (average, 123,038) were generated, indicating that the coverage of SLAFs in each progeny ranged from 2.31-fold to 3.61-fold (average, 3.11-fold; Fig. S1).

### SLAF marker detection and genotype definition

**Figure 1:**
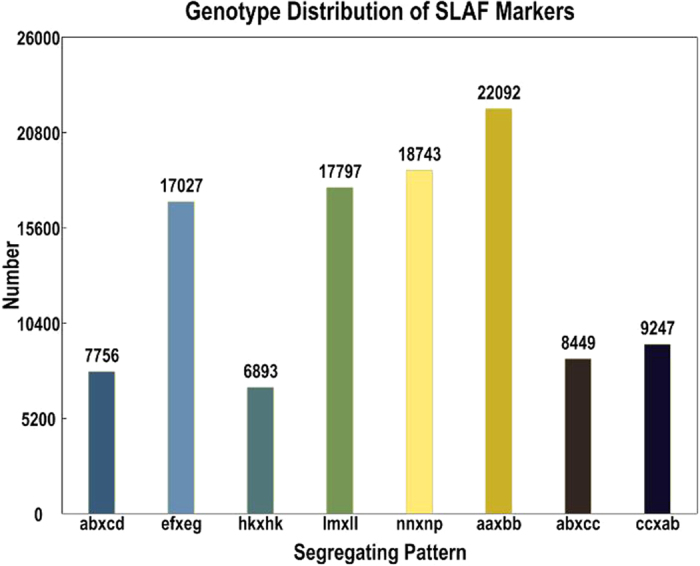
Distribution of SLAF markers in eight segregation patterns.

Among the 239,704 high-quality SLAFs, 132,542 were polymorphic with a polymorphism percentage of 55.3% (Table 1). Of the 132,542 polymorphic SLAFs, 108,004 were classified into eight segregation patterns. The segregation patterns were as follows: ab × cd, ef × eg, hk × hk, lm × ll, nn × np, aa × bb, ab × cc, and cc × ab (Fig. 1). Since the F_1_ population was used in this study, only SLAF markers with segregation patterns of ab × cd, ef × eg, hk × hk, lm × ll, nn × np, ab × cc, or cc × ab were used for the genetic map construction. To further improve SLAF accuracy, only SLAFs with more than 80% integrity and more than 20-fold average sequence depths in the parents were used for the map construction. Finally, 4,508 of the 108,004 markers were selected for the linkage map construction (Table 2). Of these 4,508 markers, 60.96% were heterozygous in the male parent; 61.78%, in the female parent; and 21.33% to 47.07%, in the F_1_ individuals. Average sequencing depths of the 4,508 markers were 86.71-fold and 92.66-fold for the male and female parents, respectively, and 7.99-fold for each progeny.

### Construction of the high-density linkage map

**Figure 2:**
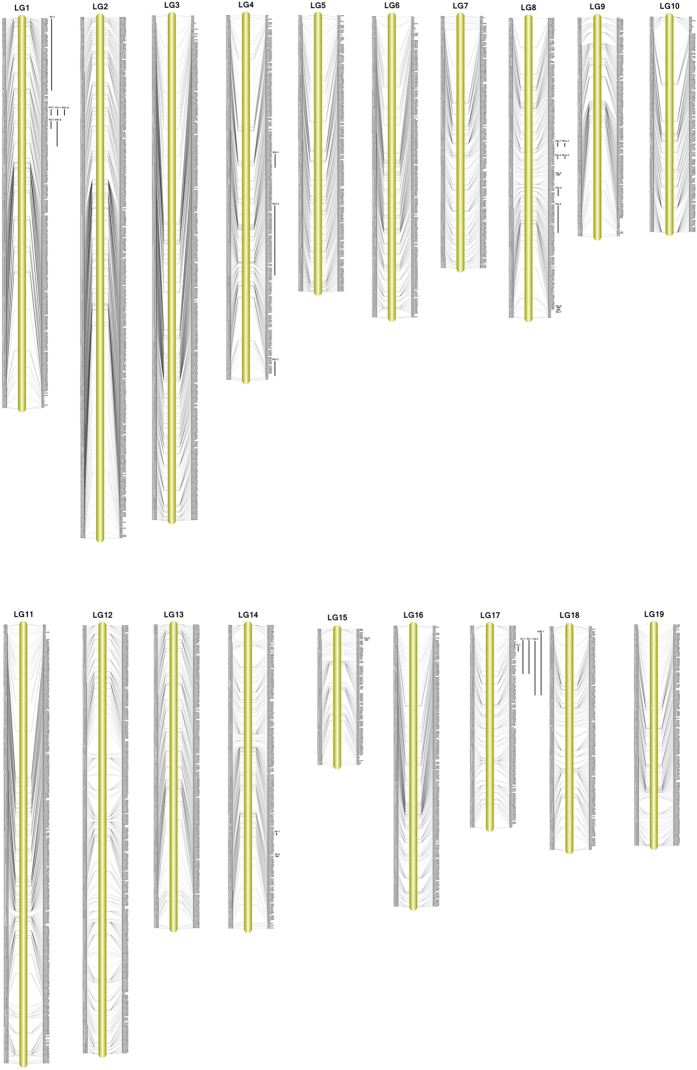
The high-density genetic map and QTLs for traits associated with pearl quality in *Hyriopsis cumingii*. The markers and their locations are shown on the right and left sides, respectively. QTLs are marked on the right of each linkage map.

To construct a high-density, high-quality genetic linkage map for *H. cumingii*, the newly developed 4,508 SNPs and 506 SSR markers from the framework map^32^ were used. After linkage analysis, 4,920 markers were mapped to 19 linkage groups (Table 3, Fig. 2). The final map was 2,713 cM in length, with an average of 79 loci per LG and an average inter-marker distance of 1.81 cM. The number of markers mapped to each linkage group varied from 114 markers in LG15 to 436 markers in LG2, with an average of 258 markers per LG. The largest LG was LG2, which contained 436 markers with a genetic length of 196.50 cM, and the smallest LG was LG10, which contained only 180 markers with a length of 91.09 cM. The mean chromosome region length was 142.80 cM. The ‘Gap ≤ 5 cM’ value (indicates the percentages of gaps in which the distance between adjacent markers is smaller than 5 cM) of the 19 linkage groups ranged from 80.28% to 100% (average, 97.79%). The maximum gap was 18.91 cM, which was observed in LG15.

In this study, sex-specific maps were also constructed (Table S4). The male map contained 3,233 markers and spanned 2,561 cM, while the female map contained 3,123 markers and spanned 2,810 cM. In addition, the male map contained 170 markers and 48 loci per LG, while the female map contained 164 markers and 56 loci per LG. The average inter-marker distances for the male and female maps were 2.78 and 2.66 cM, respectively. Although the lengths of the male and female maps were similar, we observed significant differences in some of LGs. For instance, the ratios of lengths of LG10, LG13, and LG18 in the male and female maps were 1.39, 1.45, and 1.61, respectively (Table S4).

### Comparison of the constructed map with the SSR-based framework map

Since we constructed an SSR-based framework map for *H. cumingii* in a previous study, we performed a brief comparison of the two maps. Although both maps comprised 19 linkage groups, significant differences were observed in the number of markers, map length, and resolution between the two maps. The SSR-based framework map contained only 506 SSR markers and spanned 1,922.3 cM, which is obviously less than the 4,920 markers and 2,713 cM map length observed in this study. Our current linkage map has higher resolution at 1.81 cM, which is significantly superior to the resolution of the previous genetic map with an average inter-marker distance of 3.99 cM. Besides, we noted many large gaps in the SSR-based map and a lesser number of large gaps in the current map.

### QTL mapping for pearl quality-related traits

**Figure 3:**
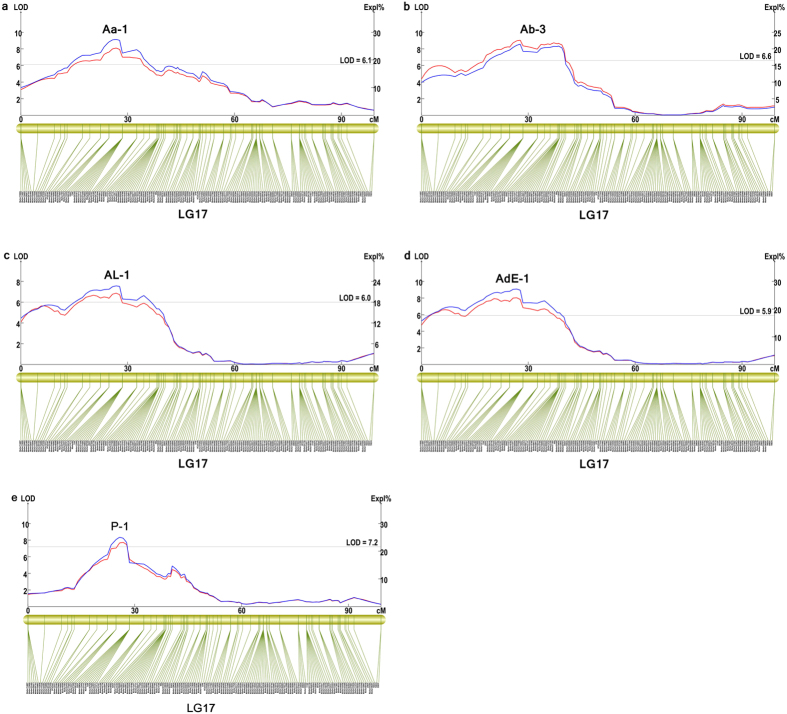
Mapping of significant QTLs for shell nacre colour. (**a**) QTL for Aa in LG17; (**b**), QTL for Ab in LG17; (**c**), QTL for AL in LG17; (**d**), QTL for AdE in LG17; (**e**), QTL for purple mantle scar length in LG17. The blue line and red line represent the LOD score and the explained phenotypic variation of QTL, respectively.

The basic statistics for the pearl quality traits are listed in Table S2. On the basis of the high-density genetic map, a total of 26 QTLs were detected on LG1, LG4, LG8, LG14, LG15, and LG17 (Table 4, Fig. 3). Phenotypic variability explained by each QTL ranged from 8.4% to 22.8% (average, 12.3%). Three QTLs for shell width were located on LG1, and the confidence intervals were at 0–11.3, 15.5, and 21.8–22.4 cM. Two QTLs for shell weight were detected at 15.5 cM on LG1 and 11.2 cM on LG15. Seven QTLs for body weight spanned discontinuously from 76.3 to 157.6 cM on LG8. For inner mantle weight, three QTLs spanned discontinuously from 107.6 to 184.5 cM on LG4, and two QTLs were at 15.5 and 21.8–27.6 cM on LG1. Besides, two QTLs for margin mantle weight were located on LG8, and the confidence intervals were at 78.6–79.3 and 82.9 cM. For the growth-related traits, three QTLs (Wi-2, Whi-4, and Sw-1) were found in the same region on LG1, and they showed a percentage of phenotypic variance explained (PVE) of 10.0%, 8.4%, and 8.8%, respectively. Two QTLs (We-2 and Who-2) were found in the same region on LG8 near Marker93674 and Marker261215, with a PVE of 9.7% and 11.8%, respectively. Additionally, seven QTLs associated with shell nacre colour were detected, showing a high percentage of PVE of 19.7% to 22.8%. Of these seven QTLs, five significant QTLs related to shell nacre colour tended to colocalize, clustering in a special region (17.3 cM to 36.1 cM) on LG17 (Fig. 3).

### The validation of four QTLs associated with shell nacre colour on LG17

Statistical analysis showed that all three markers were significantly associated with shell nacre colour (*P* < 0.05). For Marker257873, the *H. cumingii* with CC genotype showed a significantly higher value in AL while significantly lower values in Aa, Ab and AdE, compared with the individuals with CT or TT genotypes. For Marker189034, the individuals with AA genotype showed significantly higher values in Aa, Ab and AdE, but a significantly lower value in AL when compared with the individuals with GA or GG genotypes. Similarly, as for Marker273509, the individuals with GG genotype had a significantly lower value in AL but higher values in Aa, Ab and AdE (Table 5).

## Discussion

High-density linkage maps are especially important for QTL mapping, positional cloning, comparative genomics, MAS, and identification of functional genes^35^,^36^,^37^,^38^. Currently, although SNPs have become the first-selected genetic markers for high-density linkage map construction, their widespread application remains limited by the high costs of high-throughput sequencing and genotyping^39^. Fortunately, NGS-based marker identification and genotyping technologies provide a powerful method for the identification of large numbers of SNPs. The SLAF-seq approach balances genotyping accuracy and sequencing cost, thus providing an economical and efficient method for linkage mapping of non-model species with complex genomes^24^,^25^,^26^,^27^,^40^.

In this study, SLAF-seq was used to develop SLAF markers in an F_1_ full-sib family of *H. cumingii*. By SLAF library construction and high-throughput sequencing, 239,704 high-quality SLAFs were detected, and 129,967 SLAFs were polymorphic with a high polymorphism rate of 54.22%. Although the SLAF-seq strategy provides a powerful tool for large-scale SNP identification, some inevitable errors and missing values occur during SLAF marker development and genotyping^41^. Therefore, strict criteria were used to avoid false-positive markers, and 4,508 markers were finally used for the linkage mapping. Since the sequencing depth reflects the accuracy of *de novo* SNP identification and genotyping quality scores can be used to detect suspicious markers, they are both important criteria for ensuring genotyping accuracy. The minimum sequencing depth for each individual is 6-fold when the SLAF-seq approach is used^24^. In our study, average sequencing depths were 86.71-fold and 92.66-fold for the male and female parents and nearly 8-fold for each progeny, which provided a high level of genotyping accuracy. In addition, genotyping quality scores of these 4,508 markers reached 30, which effectively eliminated the suspicious markers. We excluded markers with significant segregation distortion (the genotype integrity of these markers was less than 80% in the mapping population), thus ensuring the quality of the SLAF markers and genotyping accuracy. Therefore, our SNP genotyping data can be reliably used for high-density linkage map construction.

We constructed a high-density genetic map for *H. cumingii*, containing 4,920 markers with a resolution of 1.81 cM. The high-density genetic map is a great improvement over our previous map for *H. cumingii*^32^, providing an important tool for fine mapping of QTLs for important traits. Our current linkage map is better than previous genetic maps for bivalves such as *P. maxima* (1,189 SNP markers)^42^, *Pinctada fucata martensii* (3,117 markers)^43^, and *C. farreri* (3,806 markers)^7^ with respect to marker number, but it is slightly inferior with respect to average marker interval because of the larger genome size of *H. cumingii*. We also observed some large gaps in the current map; this suggested that there may be a higher level of recombination or that markers in these regions have not been developed. Hence, other enzymes may be used for SLAF library preparation, and the size of the mapping population needs to be improved in the future.

When compared with the SSR-based framework map^32^, the map constructed in this study spanned a cumulative distance of 2,713 cM, showing a significant increase in map length. Of course, the genetic length of a linkage map could be affected by many factors, such as the number and type of markers, size of the mapping population, and accuracy of the genotyping^37^. Errors in genotyping usually cause elongation of a linkage map. In our study, strict criteria were used during SLAF marker development and genotyping, which ensured the quality of the SLAF markers and greatly reduced genotyping errors; this enhanced the accuracy of the genotyping and, thus, effectively avoided artificial inflation of map length. Therefore, elongation of the genetic map in this study was probably caused by the variation in the number and type of the markers and changes in the size of the mapping population.

Accuracy of marker order and the genetic map is one of the most interesting aspects for people who work with genetic mapping. In this study, the order of common SSR markers in the present map and the previous framework map was compared^32^. For most LGs, a roughly similar order was observed for the SSR markers; however, different orders of common SSR markers were observed in some LGs. The variation may be partly due to the different numbers of markers and individuals used for the two genetic maps. A previous study showed that a larger-sized population is considered sufficient for the construction of reasonably accurate genetic maps^44^. Furthermore, this effect will be even more obvious when the number of different families increased, since it could increase the power to map loci by increasing the number of informative meiosis events. In addition, different methods for map construction may influence the accuracy of marker order and the genetic map. Some minor differences in marker order have been observed between linkage maps constructed using JoinMap4.1 and HighMap^41^. A previous study has reported that HighMap has many advantages over JoinMap4.1^45^. HighMap not only permits the use of more NGS data but could also guarantee higher accuracy of marker order and map distance for data with a large proportion of missing or erroneous markers.

Many factors affect pearl quality, such as size, shape, colour, and lustre. Since production of a non-nucleated pearl requires the participation of both donor and host mussels, previous studies have highlighted the link between pearl traits and growth-related traits in *H. cumingii*. A previous study has shown that shell weight, body weight, and shell width are significantly associated with pearl diameter and pearl yield^46^. Margin mantle of the donor mussel is used to make the saibo, and size and thickness of the margin mantle determines the size and thickness of the saibo. The inner mantle, in which a non-nucleated pearl is implanted in the receptor mussel, directly affects pearl growth. Therefore, these five growth traits are considered related to pearl quality^47^. Pearl colour has always been an important criterion for evaluating pearl value, and shell colour is believed to have a connection with pearl colour because of their similarities in formation. A previous study revealed that pearl colour is related mainly to the shell nacre colour of the donor mussel in *H. cumingii*^48^. The results showed that extremely significant correlation existed between inner shell color of the donor mussel and pearls color (*P* < 0.01). Moreover, the greater the dE (total colour change) value of the donor mussels is, the higher percent of purple pearl the mussel can produce. Therefore, pearl colour may be improved by improving shell nacre colour in *H. cumingii*.

A high-density linkage map is an effective tool for the fine mapping of QTLs for these important traits in *H. cumingii.* In this study, shell nacre colour and growth-related traits were fine mapped using the improved linkage map, and 26 putatively significant QTLs were detected. Compared to our previous study, six more QTLs for growth-related traits were detected, and the positions of the QTLs were refined to a greater degree. However, the QTLs detected on LG1, LG4, LG8, and LG15 were significantly different from the results of our previous study^32^, in which growth-related QTLs were located on LG1, LG2, LG4, LG6, LG8, and LG18. Such inconsistent results are possibly due to the differences in the resolution of the two genetic maps. To date, one or two QTLs for some traditional growth-related traits, such as body weight, shell width, and shell weight, have been reported in many bivalves^7^,^28^,^30^,^33^,^49^. In this study, seven QTLs for body weight, three QTLs for shell width, and two QTLs for shell weight were identified, with an average PVE of approximately 10%; thus, a higher QTL number and lower PVE were observed when compared with the abovementioned studies. In addition, five QTLs for inner mantle weight and two QTLs for margin mantle weight, which were considered to be related to pearl formation, were identified in our study. Since there is little doubt that shell formation and pearl formation are inextricably linked^50^ and close ties exist between the growth traits and pearl traits, all the detected QTLs may have a potential impact on shell or pearl formation and should be investigated further. In the present study, seven QTLs associated with shell nacre colour were detected, showing a high percentage of PVE of 19.7% to 22.8%. Of these seven QTLs, five significant QTLs related to shell nacre colour tended to colocalize, clustering in a special region (17.3 cM to 36.1 cM) on LG17. This result showed a high similarity to the previously identified five QTLs located on the same chromosome in the region of 0–42.6 cM. In particular, the significant LOD region (17.3–36.1 cM) of these five QTLs contained 29 markers and showed smaller marker gaps of no more than 3.8 cM. These findings were more refined than those of our previous study in which only seven markers were found and the biggest gap was 16.2 cM (0–42.6 cM). Furthermore, three SNPs located on four QTLs were selected and validated to be significantly associated with shell nacre colour in other families, which suggested that the shell nacre colour related QTLs identified in the study had high accuracy and reliability. Although there are few studies on QTLs for shell nacre colour, a recent study on *P. maxima* reported nine putative QTLs for pearl colour and one QTL for pearl surface complexion^34^. Moreover, the most prominent QTLs were located within a 2-cM interval on LG10, which is similar to our findings on QTLs for shell nacre colour. Given the correlation between shell nacre colour and pearl colour, we believe that there are some important genes or loci that determine shell nacre colour or pearl colour on LG17. Further studies need to be performed to identify genes that determine shell nacre colour in *H. cumingii*.

## Methods

### Mapping and DNA isolation

An F_1_ full-sib family of *H. cumingii* produced by an intra-specific cross between two mussels was used as the mapping family. The female and male parents are from the Poyang Lake population and Dongting Lake population, respectively. The aquaculture environment and management process were as described by Bai *et al.*^32^. DNA was extracted from 200 F_1_ individuals and their parents, according to a method described previously^51^. Finally, parentage analysis was carried out, and paternity of 157 F_1_ progenies was confirmed using eight microsatellite markers, as described previously^52^.

### SLAF library preparation and sequencing

The SLAF library was prepared as described previously^24^, with minor modifications. A pre-designed experiment was conducted to evaluate the enzymes and restriction fragment sizes. On the basis of the results of the pilot experiment, the optimum scheme was confirmed and used to construct the SLAF library. Genomic DNA from the two parents and F_1_ progenies was incubated at 37 °C with *Mse*I (New England Biolabs, NEB), T4 DNA ligase (NEB), ATP (NEB), and *Mse*I adapter. Restriction-ligation reactions were heat-inactivated at 65 °C, and digestion was performed using *Hae*III and *Pvu*II*-HF*^*TM*^ at 37 °C. PCR was performed with a diluted restriction-ligation mixture, dNTP, *Taq* DNA polymerase (NEB), and *Mse*I primer containing barcode 1. Next, we used the E.Z.N.A. Cycle Pure Kit (Omega) to purify and pool the PCR products. The pooled PCR products were subsequently incubated at 37 °C with *MseI*, T4 DNA ligase, ATP, and Solexa adapter. Then, the sample was purified using a Quick Spin column and run on a 2% agarose gel. Subsequently, fragments ranging from 314 to 394 bp were excised and purified using a Gel Extraction Kit, and these DNA fragments were amplified using PCR with the Phusion Master Mix (NEB) and Solexa Amplification primer mix to add barcode 2. Then, DNA fragments of 314–394 bp were gel-purified and diluted for pair-end sequencing on an Illumina High-seq 2500 sequencing platform at Biomarker Technologies Corporation in Beijing.

### SLAF-seq data analysis and genotyping

SLAF-seq data analysis and genotyping were performed according to the method developed by Sun *et al.*^24^. Briefly, all SLAF pair-end reads with clear index information were clustered on the basis of sequence similarity detected using BLAT^53^. Then, sequences with more than 90% similarity were assigned to the same locus. Minor allele frequency (MAF) evaluation was subsequently performed to define alleles in each SLAF. *H. cumingii* is a diploid species, and one locus contains no more than four SLAF tags; therefore, loci containing more than four tags were discarded as repetitive SLAFs. Correspondingly, groups with two, three, and four tags were identified as polymorphic and considered potential SLAFs. To further improve SLAF accuracy, SLAFs with more than 80% integrity and more than 20-fold average sequence depths in parents were used for the map construction.

### Construction of the genetic linkage map

To reduce the influence of distorted markers, the chi-square test (χ^2^) was performed and the SLAF markers with significantly distorted segregation (*P* < 0.05) were excluded from the linkage mapping. Then, all high-quality SLAF markers and 506 SSR markers of the previous study were used for the linkage map construction^32^. The markers were divided into LGs by using the single-linkage clustering algorithm at logarithm of odds (LOD) threshold ≥4.0 and a maximum recombination fraction of 0.4. To avoid genotyping errors and deletion caused by NGS, High Map Strategy was used^45^. MSTmap algorithms^54^ and SMOOTH algorithms^55^ were used to order the SLAF markers and correct genotyping errors, respectively. The LGs were constructed as follows: Primary marker orders were first obtained by their location on the chromosomes, according to the relationship between ordered markers, and genotyping errors or deletions were corrected using the SMOOTH algorithm; then, MSTmap was used to order the map and SMOOTH was again used to correct the newly ordered genotypes. The processes were performed iteratively to ensure the accuracy of marker order and map distances, and high-quality maps were constructed after four or more cycles.

### Phenotypic measurement and QTL analysis

The following five growth-related traits were measured in the 157 progenies: shell width, body weight, shell weight, margin mantle weight, and inner mantle weight and five shell colour-related traits, purple mantle scar length, CIE 1976 L* (lightness), a* (redness), b* (yellowness) colour space, and total colour change (dE)^32^. The average L* (AL), average a* (Aa), average b* (Ab), and average dE (AdE) represent average values measured on the three points of inner shell. Statistical analysis of the phenotypic data was conducted with SPSS 18.0. MapQTL 5.0 software was used for the QTL mapping with the interval mapping method. The genome was scanned at 1-cM intervals, the maximum LOD score along the interval was considered as the position of the QTL, and the region in the LOD score greater than the threshold was considered as the confidence interval. The LOD score threshold was initially set at 3.0 for QTL declaration, and QTLs that exceeded this LOD threshold were considered as suggestive QTLs. If any relevant QTL was identified, the LOD score threshold was determined using the 1,000-permutation test with a confidence of 0.99. QTLs with LOD scores greater than the threshold at a confidence of 0.99 were declared significant.

### The validation of four QTLs associated with shell nacre colour on LG17

To examine whether the SNPs located on QTLs were associated with shell nacre colour, three SNPs were selected from shell nacre colour related QTLs (AL-1, Aa-1, Ab-3, AdE-1) and used for primer design (Table S5). Then, the primers and markers were assessed in three families of *H. cumingii* (one hundred individuals for each family) from Chongming aquaculture farm of Shanghai Ocean University in Shanghai, China. Genomic DNA was extracted and CIE 1976 L* (lightness), a* (redness), b* (yellowness) colour space, total colour change (dE) were measured as described above. Primer designing and genotyping assays were carried out using KASP technology (LGC Genomics, Hoddesdon, UK) as described previously^56^. Associations between SNPs and shell nacre colour related traits were analyzed using Chi-square test in JMP 8.0 software.

## Additional Information

**How to cite this article**: Bai, Z.-Y. *et al.* Construction of a high-density genetic map and QTL mapping for pearl quality-related traits in *Hyriopsis cumingii. Sci. Rep.* **6**, 32608; doi: 10.1038/srep32608 (2016).

## Supplementary Material

Supplementary Information

Supplementary Tables

## Figures and Tables

**Table 1:** Mining results for SLAF markers.

Type	No. of reads	No. of SLAFs	Coverage	Ratio
Polymorphic SLAF	39,152,380	132,542	295	55.3%
Non-polymorphic SLAF	29,278,441	107,162	273	44.7%
Total	68,430,821	239,704	285	100.0%

**Table 2:** Statistics for segregation types of the SLAF markers.

Type	No. of SLAFs	Percentage
abxcd	52	1.15%
efxeg	387	8.58%
hkxhk	586	13.00%
lmxll	1,723	38.22%
nnxnp	1,760	39.04%
Total	4,508	100.00%

**Table 3:** Summary of the integrated linkage map for *H. cuming*ii.

Linkage group	Marker no.			Number of loci	Size (cM)	Average distance (cM)	Gap ≤ 5	Max gap (cM)
	SSR	SNP	Total					
LG01	23	303	326	84	152.06	1.81	92.77	15.99
LG02	36	400	436	127	196.50	1.55	95.23	8.09
LG03	35	387	422	105	157.35	1.50	97.12	13.53
LG04	36	269	305	72	185.50	2.58	80.28	17.24
LG05	29	202	231	81	152.51	1.88	95.00	15.34
LG06	33	219	252	82	155.03	1.89	91.36	14.60
LG07	27	184	211	64	102.17	1.60	95.24	14.71
LG08	24	227	251	86	168.70	1.96	92.94	16.44
LG09	24	159	183	65	124.08	2.00	90.63	13.26
LG10	35	145	180	47	91.09	1.94	93.48	16.21
LG11	27	340	367	98	176.70	1.80	92.78	17.56
LG12	21	337	358	85	102.09	1.20	100.00	4.91
LG13	11	243	254	89	121.64	1.37	96.60	8.73
LG14	24	230	254	89	151.57	1.70	95.45	10.55
LG15	23	91	114	47	139.16	2.96	89.13	18.91
LG16	23	212	235	82	165.53	2.04	90.12	12.61
LG17	15	154	169	62	99.13	1.60	98.36	7.55
LG18	14	173	187	54	137.82	2.55	90.91	11.00
LG19	15	170	185	81	134.55	1.66	93.75	10.07
Total	475	4,445	4,920	1,500	2,713.17	/	/	/
Average	25	234	259	79	142.80	1.81	93.46	/

**Table 4:** Summary of QTLs for pearl quality-related traits in F_1_ populations of *H. cuming*ii.

Trait	QTL ID	LOD threshold	LG group	QTL interval (cM)	Peak position (cM)	Peak LOD value	PVE (%)		Marker number
							Minimum	Maximum	
Shell width	Wi-1	3.0	LG1	0–11.3	1.6	3.84	9.1	10.7	62
	Wi-2	3.0	LG1	15.5	15.5	3.58	10.0	10.0	6
	Wi-3	3.0	LG1	21.8–22.4	21.8	3.14	8.5	8.8	7
Body weight	We-1	3.0	LG8	76.3–79.6	79.6	3.25	8.4	9.1	4
	We-2	3.0	LG8	82.9	82.9	3.47	9.7	9.7	2
	We-3	3.0	LG8	89.2	89.2	3.06	8.8	8.8	1
	We-4	3.0	LG8	95.1	95.1	3.31	9.2	9.2	6
	We-5	3.0	LG8	99.6–105.7	100.9	3.60	9.3	10.0	23
	We-6	3.0	LG8	155.6	155.6	3.35	9.5	9.5	1
	We-7	3.0	LG8	157.6	157.6	3.04	8.5	8.5	1
Shell weight	Sw-1	3.0	LG1	15.5	15.5	3.12	8.7	8.8	6
	Sw-2	3.0	LG15	11.2	11.2	3.12	8.7	8.7	1
Inner mantle weight	Whi-1	3.0	LG4	107.6–108.2	108.2	3.04	8.4	8.5	12
	Whi-2	3.0	LG4	120.9–126.1	125.8	3.27	8.5	9.8	59
	Whi-3	3.0	LG4	148.0–184.5	167.8	3.76	8.6	10.4	13
	Whi-4	3.0	LG1	15.5	15.5	3.01	8.4	8.4	6
	Whi-5	3.0	LG1	21.8–27.6	27.6	3.22	8.4	9.2	22
Margin mantle weight	Who-1	4.1	LG8	78.6–79.3	78.6	4.25	11.4	11.7	2
	Who-2	4.1	LG8	82.9	82.9	4.28	11.8	11.8	2
Purple mantle scar length	P-1	7.2	LG17	24.7	24.7	8.06	21.0	21.1	5
AL	AL-1	6.0	LG17	17.3–36.1	28.5	7.39	16.5	19.7	29
Aa	Aa-1	6.1	LG17	17.3–36.1	28.5	8.77	16.4	22.8	29
Ab	Ab-1	6.6	LG14	79.6	79.6	6.72	17.9	17.9	2
	Ab-2	6.6	LG14	85.0	85.0	6.72	17.9	17.9	1
	Ab-3	6.6	LG17	19.4–40.1	28.5	8.55	18.6	22.2	45
AdE	AdE-1	5.9	LG17	4.7–40.1	24.8	8.79	15.9	22.8	52

**Table 5:** Associations between three SNPs and shell nacre colour related traits in *H. cumingii*.

SNP	Genotype	No.	AL	Aa	Ab	AdE
Marker189034	AA	86	49.81 ± 5.36^a^	4.48 ± 2.47^a^	−6.30 ± 4.41^a^	51.96 ± 5.18^a^
	GA	144	52.38 ± 6.33^b^	3.48 ± 2.98^b^	−7.70 ± 4.18^b^	49.23 ± 6.33^b^
	GG	48	52.64 ± 5.38^b^	3.81 ± 2.39A^b^	−8.64 ± 4.48^b^	49.12 ± 5.24^b^
*P*-value			0.0029^*^	0.0274^*^	0.0064^*^	0.0015^*^
Marker257873	CC	85	53.98 ± 5.71^a^	2.92 ± 2.40^a^	−8.60 ± 4.72^a^	47.74 ± 5.60^a^
	CT	119	50.89 ± 6.19^b^	4.21 ± 2.70^b^	−7.17 ± 4.01^b^	50.75 ± 6.07^b^
	TT	68	50.33 ± 5.36^b^	4.02 ± 2.81^b^	−6.35 ± 4.60^b^	51.37 ± 5.44^b^
*P*-value			0.0001^*^	0.0020^*^	0.0055^*^	0.0001^*^
Marker273509	AA	15	55.50 ± 4.41^a^	0.85 ± 0.84^a^	−8.82 ± 3.83^ab^	45.69 ± 4.19^a^
	GA	27	56.97 ± 3.60^a^	1.15 ± 1.24^a^	−9.30 ± 4.30^a^	44.58 ± 3.32^a^
	GG	239	50.77 ± 5.86^b^	4.33 ± 2.63^b^	−7.10 ± 4.47^b^	50.97 ± 5.74^b^
*P*-value			0.0001^*^	0.0001^*^	0.0235*	0.0001^*^
