# Supplementary material for: Construction of a high-density genetic map and QTL mapping for pearl quality-related traits in Hyriopsis cumingii
Source: Sci Rep. 2016 Sep 2;6:32608. doi: 10.1038/srep32608 (PMC5009340; doi:10.1038/srep32608)
Supplement: Supplementary Information [file srep32608-s1.pdf]

Construction of a high-density genetic map and QTL mapping for pearl quality-related traits in *Hyriopsis cumingii*

Zhi-Yi Bai[a,c†], Xue-Kai Han[a†], Xiao-Jun Liu[a], Qing-Qing Li[a], Jia-Le Li[a,b,c*]

[a]Key Laboratory of Freshwater Aquatic Genetic Resources, Shanghai Ocean University, Ministry of Agriculture, Shanghai 201306, China

[b]Aquaculture Division, E-Institute of Shanghai Universities, Shanghai Ocean University, Shanghai 201306, China

[c]Shanghai Engineering Research Centre of Aquaculture, Shanghai Ocean University, Shanghai 201306, China

†These authors contributed equally to this study.

[*]Corresponding author: **Jia-Le Li**, Key Laboratory of Freshwater Aquatic Genetic Resources, Shanghai Ocean University, Ministry of Agriculture, Shanghai 201306, China

Supplementary information

Supplementary figure legends

Figure S1. Coverage and number of markers for each F_1 individual and parent. The x-axes in both **A** and **B** indicate individual accessions, including those of the female and male parents and followed by 157 F_1 progenies. The y-axes indicate coverage in **A** and number of markers in **B**.

Figure S1.

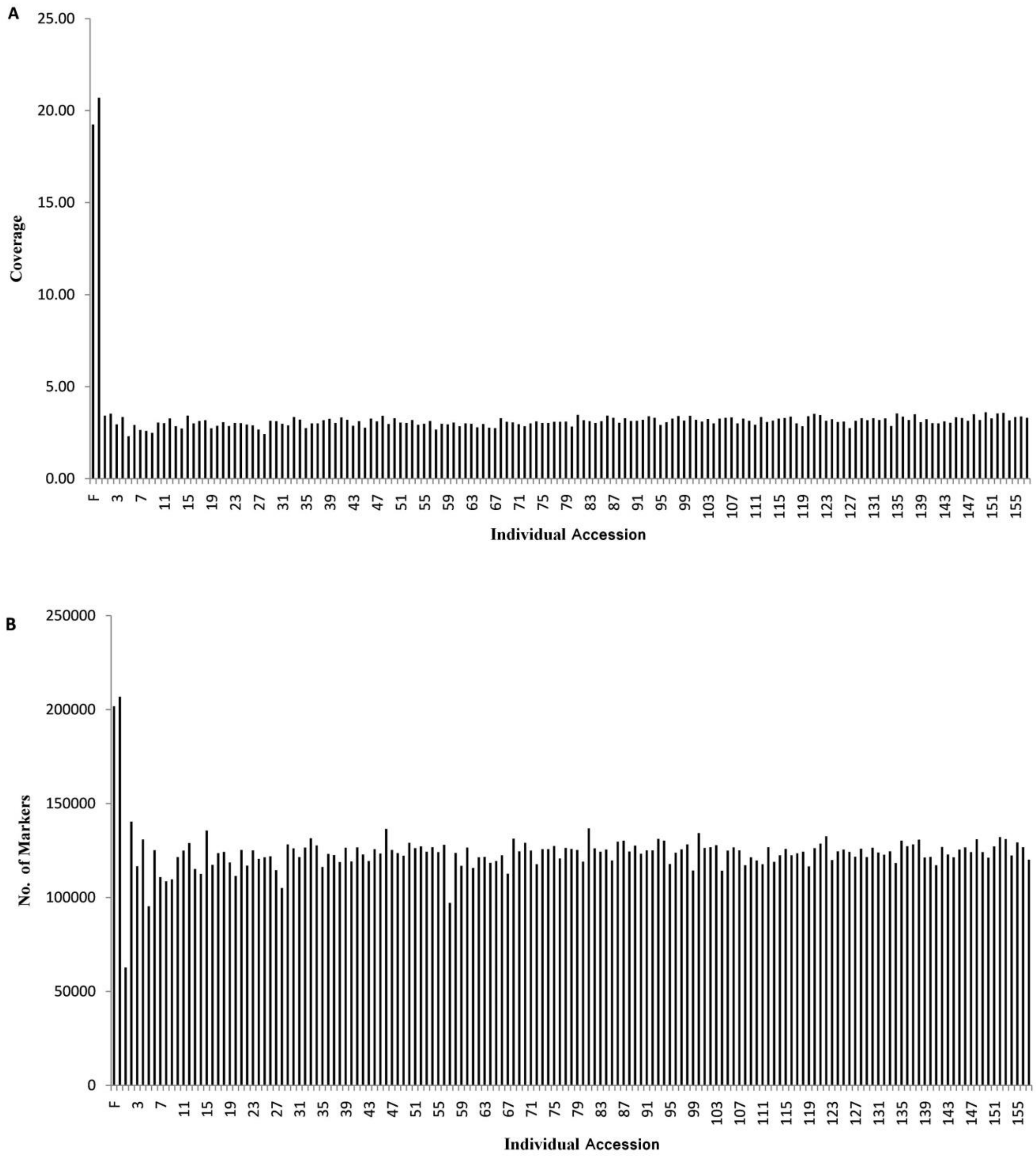

Table S2. Statistics of pearl quality-related traits of the QTL mapping family in *H. cumingii*

Traits	Range	Mean ± SD
Shell width	1.12–2.44	1.77 ± 0.24
Body weight	12.43–65.42	34.51 ± 10.88
Shell weight	4.27–20.73	11.42 ± 3.76
Margin mantle weight	0.23–0.96	0.49 ± 0.14
Inner mantle weight	0.15–1.15	0.62 ± 0.25
Purple mantle scar length	1.67–9.22	4.59 ± 1.69
AL	33.71–60.29	50.00 ± 4.48
Aa	2.88–11.23	6.66 ± 1.69
Ab	-22.79–9.23	-8.69 ± 6.20
AdE	38.63–66.57	48.51 ± 4.51

AL, average lightness; Aa, average redness; Ab, average yellowness; AdE, average total colour change.

Table S4. Summary of male and female linkage maps for *H. cumingii*.

Linkage group	Male			Female			Average distance (cM)	
	Marker no.	Locus no.	Size (cM)	Marker no.	Locus no.	Size (cM)	Male	Female
LG01	187	49	137.62	225	62	166.50	2.81	2.69
LG02	290	87	199.40	270	87	192.97	2.29	2.22
LG03	280	64	153.28	249	75	161.43	2.40	2.15
LG04	210	44	186.81	184	45	184.18	4.25	4.09
LG05	157	65	154.61	150	48	150.41	2.38	3.13
LG06	138	51	158.68	175	60	146.70	3.07	2.45
LG07	145	45	93.33	130	44	111.01	2.07	2.52
LG08	157	48	133.90	175	65	187.05	2.79	2.88
LG09	116	35	117.19	134	53	121.22	3.35	2.29
LG10	210	31	103.12	65	26	73.97	3.33	3.61
LG11	145	49	163.53	254	64	156.87	3.34	2.45
LG12	225	54	97.40	211	59	106.78	1.80	1.81
LG13	163	50	96.13	159	67	139.00	1.92	2.07
LG14	145	44	156.88	170	68	136.56	3.57	2.01
LG15	187	31	123.36	57	32	153.66	3.98	4.80
LG16	164	52	148.25	131	54	179.54	2.85	3.32
LG17	116	50	112.67	111	40	121.93	2.25	3.05
LG18	121	30	105.54	118	40	170.10	3.52	4.25
LG19	77	41	119.11	155	66	149.98	2.91	2.27
Total	3,233	920	2,560.81	3,123	1055	2,809.85	/	/
Average	170	48	134.78	164	56	147.89	2.78	2.66

Table S5 Allele-specific primers and the common primers sequence of three SNP markers.

ID	Allele_Primer_FAM (5'–3')	Allele_Primer_Hex (5'–3')	Common_Primer (5'–3')
Marker189034	CGCTCCTGGCGAGGAAGTCA	GCTCCTGGCGAGGAAGTCG	GGGAGTATAGAGGGTATACAAAGCTTT
Marker257873	TTCAGAAATTTGAAAGTGAGTCAAATCA	TCAGAAATTTGAAAGTGAGTCAAATCG	ATAGCTAAAGAGTGTCAGTGCATGACTAT
Marker273509	CTCGACAAATCGGCTGGTCAAA	CTCGACAAATCGGCTGGTCAAG	CAAGTAGATCAGACCTCCTAGAGCTT

The pairs of allele-specific primers are in the columns denoted as Allele_primer_FAM and Allele_Primer_HEX
